# Adults with Down syndrome have reduced cardiac response after light exercise testing

**DOI:** 10.1007/s12471-012-0254-1

**Published:** 2012-02-14

**Authors:** J. C. Vis, H. A. C. M. De Bruin-Bon, B. J. Bouma, S. A. Huisman, L. Imschoot, K. van den Brink, B. J. M. Mulder

**Affiliations:** 1 Department of Cardiology, Academic Medical Centre, Meibergdreef 9, 1105 AZ Amsterdam, the Netherlands; 2 Prinsenstichting, Residential Centre for People with Intellectual Disabilities, postbus 123, 1440 AC Purmerend, the Netherlands; 3 ASVZ, Residential Centre for People with Intellectual Disabilities, Postbus 121, 3360 AC Sliedrecht, the Netherlands; 4 ‘s Heeren Loo, Residential Centre for People with Intellectual Disabilities, Postbus 1035, 2680 BA Monster, the Netherlands

**Keywords:** Down syndrome, Cardiac response, Cardiac index, Exercise testing, Echocardiography, Stroke volume

## Abstract

**Objective:**

Physical fitness is reduced in adults with Down syndrome (DS). The present study was conducted to elucidate the exercise response in adults with DS.

**Design:**

Case controlled before-after trial.

**Setting:**

Residential centre for people with intellectual disabilities.

**Participants:**

96 Adults with DS, 25 non-DS adults with an intellectual disability, 33 controls.

**Interventions:**

Echocardiography to exclude heart defects and to measure cardiac index (CI) in the supine position, supine position with raised legs, and following ten knee bends.

**Main outcome measure:**

Exercise testing

**Results:**

At rest, mean CI was not significantly different between persons with DS and controls (2.3 vs. 2.4 l/min/m^2^, *p* = 0.3). However, mean CI after exercise was significantly lower in DS (2.9 vs. 3.7 l/min/m^2^, *p* < 0.001) and mean CI increase from rest to exercise was more than 50% lower in DS. On the contrary, CI after exercise was similar among controls and non-DS adults with an intellectual disability. Significantly lower stroke volumes in DS were found with insufficient heart rate response.

**Conclusions:**

CI at rest was similar in adults with DS and controls; however persons with DS have a diminished cardiac response to exercise. Stroke volumes were significantly lower in DS during exercise and a compensated heightened heart rate was absent.

## Introduction

Average life expectancy of adults with Down syndrome (DS) has increased from a mere 12 years in the 1940s to 60 years in present-day populations [1–4]. This is due to improved techniques and outcome of corrective cardiac surgery and available advanced therapies for DS patients [5, 6]. However, DS is associated with a high prevalence of comorbid conditions, such as osteoarthritis, obesity and Alzheimer’s disease at relatively young age [7–11]. Therefore there has been growing attention for the prevention of conditions that lead to participation limitations and inactivity.

Lower levels of cardiovascular fitness have been reported previously in children and adults with DS compared with subjects without DS with or without an intellectual disability [12–14]. Also aerobic capacity exhibits a different age-related response in persons with DS as relative VO2 peak does not decline with age [15]. A combination of chronotropic incompetence [16, 17] and sympathetic dysfunction [18] may explain the very low maximal heart rate and could contribute to the reduction in aerobic power in persons with DS. Hypothetically, exercise programs could have a positive affect on overall health and lead to an increased quality of life in adults with DS. Most training programs among adults with DS did not yield the desired response of improved cardiovascular capacity [19, 20], although Tsimaras et al. found improvement of peak aerobic capacity in 25 adults with DS following a jog-walk training program [21]. We hypothesised that cardiac response to exercise would be lower in patients with DS. The primary goal of this study was to further elucidate the physiological response to physical activity in healthy adults with DS.

## Methods

### Study population

We included adults with an intellectual disability with and without DS, living in health care institutions (group homes) for people with intellectual disabilities. A control group of healthy non-athletic volunteers was randomly sampled from the Academic Medical Centre in Amsterdam (including hospital employees and medical students). Exclusion criteria were: 1) congenital heart defects 2) Alzheimer’s disease 3) contraindications to exercise or physical incompetence to perform the exercise protocol (wheelchair user, orthopaedic injury) 4) unregulated thyroid function 5) patients without sinus rhythm. An electrocardiogram was taken to examine arrhythmias and thyroid function was checked in the participants with DS by medical charts according to the latest laboratory testing within 1 year. Approval was obtained from ethics boards of all participating institutions and informed consent was acquired from all subjects and/or their legal guardians

### Echocardiography

An echocardiogram was performed in all adult patients with a portable GE VIVID I (Horten, Norway), by an experienced ultrasound technician and evaluated by a cardiologist. Echocardiography was performed to exclude congenital heart defects and standard echo measures were obtained in the supine position, supine position with raised legs (in 45° angle to increase preload), and in the supine position following ten knee bends. All echocardiographic images were acquired according to recommendations of the American Society of Echocardiography [22, 23], recorded digitally, and analysed offline. The following echocardiographic dimensions were measured: left ventricular end-diastolic diameter (*LVEDD*), left ventricular outflow tract diameter (*LVOTd*), in parasternal short-axis view, velocity time integral (*VTI*) in apical long-axis view by pulsed wave (PW) Doppler. The PW sample volume was positioned just proximal to the aortic valve leaflets, within the LVOT. The VTI was measured by tracing the leading edge of the velocity spectrum. Cardiac output was calculated using the following formulas: *Area (A) = (LVOTd/2)* ^*2*^ × *π*; *stroke volume (SV) = A* × *VTI; CO = SV* × *heart rate*. Cardiac output was adjusted for body surface area to obtain cardiac index (CI).

### Exercise test

After echocardiographic measurements in supine position and in supine position with raised legs, patients had to perform a simple test. First, the patient was informed about the test procedure. Subsequently patients had to stand next to the examination bed and were asked to perform ten knee bends (without touching the legs or knees with the arms). Immediately after this task, patients had to lie on the examination bed in a supine position and echocardiographic measurements were repeated. The total duration of the task and the delay between the end of task and echocardiographic recordings were measured with a stopwatch. Task duration of less than 45 s was accepted. Patients who did not meet this criterion were excluded as their task performance was inadequate.

### Statistical analysis

Descriptive statistics were used to describe baseline characteristics. Patient characteristics were compared between groups using chi-squared test in case of categorical variables whereas continuous variables were compared using one-way analysis of variance (ANOVA). Repeated measures ANOVA (Group X Condition) were applied to compare continuous variables among the three groups at rest, in a supine position with raised legs and after exercise. If Mauchly’s test indicated that the assumption of sphericity had been violated, degrees of freedom were corrected using the Huynh-Feldt correction. Data are given as mean ± standard deviation (SD) and the level of significance was set at *p* < 0.05. A multiple linear regression model for cardiac index was done to adjust for baseline differences between groups. Those variables found to be significant by univariate analysis (*p* < 0.1) were put in the multivariate model. Statistical analysis was performed with the SPSS software for Windows XP version 16.0.

## Results

### Study population

In total, 96 adults with DS (mean age 42 ± 11 years, 48% males), 25 non-DS adults with an intellectual disability (mean age 50 ± 11 years, 60% males) and 33 controls (mean age 40 ± 11 years, 55% males) participated in this study. Baseline characteristics of all subject groups are shown in Table 1. Cardiac output testing at rest could be performed in all 154 subjects. However cardiac index after exercise could only be obtained from 52 DS patients, 11 non-DS adults with an intellectual disability and 22 controls (49% of the included population). In the two groups with an intellectual disability, the patients with a severe cognitive impairment had a failed exercise test significantly more often (DS *p* < 0.001 and non-DS *p* = 0.04). All other baseline characteristics of patients with and without exercise testing were not significantly different, except for the non-DS adults with an intellectual disability; patients with a successful exercise test were on average 13 kg heavier. Mean time of exercise testing was 24 ± 6 s and mean delay between exercise testing and echocardiographic recording was 32 ± 13 s. In total, 54 DS persons and 14 non-DS adults with an intellectual disability were excluded for exercise testing, because they were not able to execute the exercise test adequately or did not want to participate.

**Table 1:** Baseline characteristics

	Intellectual disability		Controls	*p*-value
	Down syndrome	Non-Down-syndrome		
	*n* = 96	*n =* *25*	*n* = 33	
Male gender (%)	48	60	55	NS
Age, *yrs*	42 ± 11	50 ± 11	40 ± 11	0.002^a,b^
Weight, *kg*	64 ± 11	77 ± 15	72 ± 12	<0.001^b,c^
Height, *cm*	153 ± 8	168 ± 13	177 ± 9	<0.001^a,b,c^
BMI, *kg/m* ^*2*^	27 ± 4	27 ± 4	23 ± 3	<0.001^a,c^
BSA, *m* ^*2*^	1.6 ± 0.2	1.9 ± 0.2	1.9 ± 0.2	<0.001^b,c^
Systolic blood pressure, *mm Hg*	115 ± 13	127 ± 12	115 ± 11	<0.001^a,b^
Diastolic blood pressure, *mm Hg*	73 ± 8	81 ± 11	77 ± 10	<0.001^b^
Intellectual disability				
Mild	3	24	NA	
Moderate	62	32	NA	
Severe	28	44	NA	
Profound	7	0	NA	
Hypertensive^d^ (%)	1	8	0	N.S
Obesitas^e^ (%)	22	20	3	N.S
Regulated hypothyroidism (%)	30	5	3	<0.05^a,c^
Regulated hyperthyroidism (%)	4	0	0	N.S

Values expressed as mean ± SD, *BMI* body mass index, *BSA* body surface area, *NA* not applicable, *NS* non significant

^a^Control versus non-Down syndrome patients with intellectual disability

^b^Down syndrome patients versus non-Down syndrome patients with intellectual disability

^c^Control versus Down syndrome patients

^d^Criterium blood pressure>140/90 mmHg

^e^Criterium BMI>30 kg/m^2^

### Cardiac indices

**Fig. 1:**
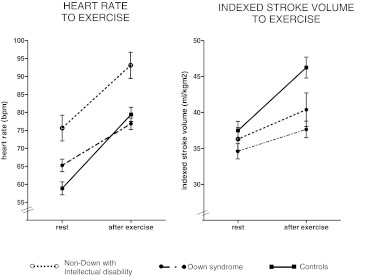
Stroke volume and heart rate response after exercise. Caption: Rest values correspond only with the subjects who performed the exercise test

**Fig. 2:**
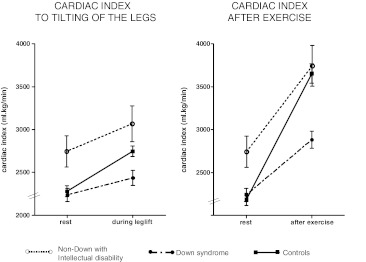
Cardiac index response. a) Cardiac index response after upright tilting of the legs. b) Cardiac index response after exercise. Caption: Rest values correspond only with the subjects who performed the exercise test

CI was not significantly different between adults with DS and controls at rest (2.3 vs 2.4 l/min/m^2^, *p* = 0.3) (Table 2). Even so, during increased preload, measured by raised legs (2.5 vs 2.7 l/min/m^2^, *p* = 0.07) cardiac index was similar. However, CI after exercise was significantly lower in DS persons (2.9 vs. 3.7 l/min/m^2^, *p* < 0.001). In controls, CI increased 69% from baseline to after exercise (*p* < 0.001). A lower CI increase of 30% was found in DS patients (*p* < 0.001). Heart rate response was comparable between DS persons and controls; however, a significantly lower stroke volume was observed in DS persons (*p* < 0.001), as shown in Fig. 1. Mean cardiac indices of all three groups after upright tilting of the legs and exercise are shown in Fig. 2. LVEDD was 40.1 ± 4.4 mm in DS persons, 42.9 ± 4.5 mm in non-DS adults with an intellectual disability and 50.0 ± 3.9 mm in controls (*p* < 0.001). By multivariate analysis, independent predictors for absolute increase of cardiac index after exercise were age (β = −0.21, *p* = 0.009), left ventricular end-diastolic diameter (β = 0.33, *p* = 0.009) and DS (β = −0.46, *p* = 0.001) (Table 3).

**Table 2:** Cardiac responses

	Intellectual disability		Controls	*p-*value
	Down syndrome	Non-Down-syndrome		
	*n* = 96	*n* = 25	*n* = 33	
Rest				
Cardiac output (l/min)	3.7 ± 0.9	5.3 ± 1.3	4.5 ± 1.0	<0.001^a,b,c^
Cardiac index (l/min/m^2^)	2.3 ± 0.5	2.8 ± 0.6	2.4 ± 0.4	<0.001^a,b,c^
Stroke volume (ml)	55.1 ± 13.6	68.3 ± 13.1	69.8 ± 11.8	<0.001^a,c^
Indexed stroke volume (ml/m^2^)	33.6 ± 7.5	36.4 ± 5.9	37.3 ± 5.6	0.02^a^
Heart rate (beats/min)	68 ± 12	78 ± 13	64 ± 12	<0.001^b,c^
Tilting of the legs^d^				
Cardiac output (l/min)	4.0 ± 1.1	6.1 ± 1.5	5.0 ± 0.8	<0.001^a,b,c^
Cardiac index (l/min/m^2^)	2.5 ± 0.7	3.1 ± 0.7	2.7 ± 0.3	<0.01^c^
Stroke volume (ml)	58.1 ± 15.0	78.8 ± 12.5	69.9 ± 10.4	<0.001^a,c^
Indexed stroke volume (ml/m^2^)	35.7 ± 7.9	39.5 ± 6.2	38.0 ± 5.9	NS
Heart rate (beats/min)	70 ± 11	77 ± 9	72 ± 10	NS
After exercise^e^				
Cardiac output (l/min)	4.7 ± 1.3	7.5 ± 1.7	6.8 ± 1.3	<0.001^a,c^
Cardiac index (l/min/m^2^)	2.9 ± 0.7	3.7 ± 0.8	3.7 ± 0.5	<0.001^a,b,c^
Stroke volume (ml)	61.8 ± 15.1	80.3 ± 13.3	85.5 ± 14.8	<0.001^a,c^
Indexed stroke volume (ml/m^2^)	37.7 ± 7.5	40.4 ± 7.7	46.3 ± 6.8	<0.001^a^
Heart rate (beats/min)	77 ± 10	93 ± 12	79 ± 9	<0.001^a,b,c^

*NS* non significant

^a^Control versus Down syndrome patients

^b^Control versus non-Down syndrome patients with intellectual disability

^c^Down syndrome patients versus non-Down syndrome patients with intellectual disability

^d^Performed in 52 Down syndrome patients and 22 controls, 11 non-Down syndrome patients with intellectual disability

^e^Performed in 42 Down syndrome patients and 22 controls, 11 non-Down syndrome patients with intellectual disability

**Table 3:** Multivariate analysis for cardiac index change (rest to exercise)

	Univariate	*P* value	Multivariate	*P* value
	Beta		Beta	
Age	−0.21	0.07	−0.24	0.009
Male gender	0.05	0.7		
BMI	−0.07	0.6		
Groups				
Controls	0.59	<0.001	–	–
Down syndrome	−0.57	<0.001	−0.46	0.001
Non Down syndrome with ID	0.05	0.7	−0.13	0.2
LV end diastolic diameter	0.62	<0.001	0.33	0.009

*BMI* body mass index, *ID* intellectual disability, *LV* left ventricular

## Discussion

The main finding of this study is that in healthy adults with DS, an increase in cardiac index is diminished after relatively light exercise test compared with healthy controls and non-DS adults with an intellectual disability. Mean increase of cardiac index after a simple exercise task was 50% lower in individuals with DS compared with healthy controls. This diminished cardiac response after exercise was related to a lower mean stroke volume in DS as no group differences for heart rate were found. In our previous study, we already showed that left ventricular volumes are smaller in patients with an intellectual disability (with and without DS) compared with healthy controls [24]. This indicates that stroke volumes are restricted to the smaller hearts in persons with DS. Furthermore, an expected higher heart rate response to compensate for the lower stroke volume was absent in persons with DS. This fits in with the theory of Baynard et al. that peak heart rate is low in individuals with DS [16]. Fernhall et al. suggested that a reduced peak heart rate and low work capacities in individuals with DS could be explained by a diminished catecholamine response to peak exercise [18]. Furthermore, Figueroa et al. reported a reduced heart rate and blood pressure response to sympathetic tasks due to blunted vagal withdrawal and reduced sympathoexcitation [25]. In addition to this reduced sympathicomimetic ability to exercise we demonstrated a reduced stroke volume in adults with DS. The findings in non-DS adults with an intellectual disability show that our results are specific to Down syndrome. In this study, a lower increased heart rate after exercise was found in persons with DS compared with non-DS adults with an intellectual disability. This corresponds to the study by Pitetti et al. where individuals without DS with an intellectual disability had a significantly higher mean heart rate than individuals with DS during treadmill walking [26]. Pitetti et al. also found a significantly higher peak VO2 in persons with an intellectual disability without DS [26]. In our study, cardiac index after exercise was similar among healthy controls and non-DS adults with an intellectual disability. Overall, we can conclude that non-DS adults with an intellectual disability are capable of increasing their cardiac capacity adequately, unlike individuals with DS.

A limitation of this study is that exercise testing failed in more than half of the included subjects with an intellectual disability. As expected most subjects with a severe intellectual disability were not able to perform the test due to comorbid physical problems or non-cooperation. However, all other important baseline characteristics were not statistically different from patients with or without successful exercise testing. Secondly, a familiarisation protocol was not carried out. However, by using a relatively simple task, which most adults with an intellectual disability could understand, we minimised possible result errors by inadequate performance of the task. Thereby, patients with inadequate testing (task duration of more than 45 s) were excluded for analysis of cardiac output change.

## Conclusion

Cardiac response to exercise is diminished in adults with DS. Stroke volumes were significantly lower in DS during exercise and a compensated increased heart rate was absent. An adequate cardiac response seems to exist in non-DS adults with an intellectual disability. A reduced cardiac response could be clinically relevant when considering that cardiac reserve may be inadequate in situations where higher cardiac output is needed, for example during fever and for sport activities. Also the effect of training programs to increase exercise capacity in the DS population could be negatively influenced when adults with DS are less able to increase their cardiac index while exercising.
